# An Effective Diagnostic Approach for Successful Recovery from *Mycobacterium avium* Systemic Infection in a Basset Hound Dog

**DOI:** 10.3390/vetsci13101045

**Published:** 2026-10-05

**Authors:** Simona Zoppi, Valeria D’Errico, Alessia Di Blasio, Pier Luigi Acutis, Marzia Pezzolato, Elena Bozzetta, Maria Angelillo, Maria Goria

**Affiliations:** 1 Istituto Zooprofilattico Sperimentale del Piemonte, Liguria e Valle d’Aosta, 10154 Turin, Italy; simona.zoppi@izsplv.it (S.Z.); pierluigi.acutis@izsplv.it (P.L.A.); marzia.pezzolato@izsplv.it (M.P.); elena.bozzetta@izsplv.it (E.B.); maria.angelillo@izsplv.it (M.A.); maria.goria@izsplv.it (M.G.); 2 S.C. Sanità Animale, Servizio Veterinario ASLTO5, Chieri, 10023 Turin, Italy; derrico.valeria@aslto5.piemonte.it

**Keywords:** avian tuberculosis, Basset Hound dog, *Mycobacterium avium* subspecies *hominissuis*

## Abstract

**Simple Summary:**

A clinical case of Avian Tuberculosis in a Basset Hound dog, caused by *Mycobacterium avium* subsp. *hominissuis*, was described, with emphasis on treatment and follow-up through to recovery. An early and rapid diagnosis, supported by molecular analysis and MIC determination, led to prompt treatment with clarithromycin (5 mg/kg orally every 12 h) and moxifloxacin (10 mg/kg orally every 24 h), resulting in complete recovery.

**Abstract:**

We describe the diagnostic pathway, clinical follow-up and resolution of generalized avian tuberculosis in a Basset Hound dog. Lethargy, anorexia, weight loss and shifting lameness were observed. Cytological examination of specimens collected from prescapular, retromandibular and popliteal lymph nodes revealed the presence of acid-fast bacilli, subsequently identified as *Mycobacterium avium* subsp. *hominissuis* by microbiological and molecular tests. MIC determination was performed to guide therapy, which was subsequently based on clarithromycin (5 mg/kg orally every 12 h) and moxifloxacin (10 mg/kg orally every 24 h). Early and rapid diagnosis, combined with prompt initiation of appropriate antibiotic treatment, resulted in complete recovery of the affected dog.

## 1. Introduction

*Mycobacterium avium* complex (MAC) comprises a group of slow-growing microorganisms characterized by varying degrees of pathogenicity, a broad host range, including humans [1,2], and widespread environmental distribution [3].

Many aspects related to MAC infection in dogs are still poorly understood, and MAC infection is sporadically reported worldwide, possibly due to the lower susceptibility of dogs compared with other species [4,5,6,7,8]. Some breeds, such as Dwarf Schnauzer and Basset Hound, are reported to be more susceptible [9,10,11,12], and, especially in the latter breed, a defect in cell-mediated immunity has been proposed [11].

More specifically, *Mycobacterium avium* subsp. *hominissuis* (MAH) is the most representative member of MAC and is recognized as an etiological agent of avian tuberculosis in both animals and humans [2]. It may represent a serious health concern, especially for immunocompromised patients [13,14].

MAH infection in carnivores may be localized to the skin [8], causing mostly local granulomas, or it may progress into a disseminated disease involving the alimentary and/or the respiratory tract [2,15]. In generalized forms, the spleen and liver, in association with lymph nodes, are commonly enlarged, and the involvement of these organs is often associated with a poor prognosis in dogs [4,5,7,9,10,11,12]. Rapid diagnosis and early treatment may improve recovery rates in affected pets and reduce zoonotic risk, although transmission from animals to humans is relatively rare [13,16].

This work represents a case report of generalized avian tuberculosis in a Basset Hound and, to the best of the authors’ knowledge, documents a rare instance of clinical recovery, achieved through an integrated diagnostic protocol and a prompt, susceptibility-guided therapeutic approach.

## 2. Materials and Methods

**Medical examinations, follow-up and sample collection.** An intact 11-month-old male Basset Hound, living in a private estate located near a chicken farm, was referred to a veterinary facility with clinical symptoms such as intermittent lethargy, anorexia, weight loss and shifting lameness that had been present for several months. The clinical history also included repeated administration of enrofloxacin without significant clinical improvement. Given the clinical presentation of generalized lymphadenopathy, differential diagnoses initially included multicentric lymphoma, vector-borne infections (*Leishmania infantum*, *Ehrlichia* spp., *Anaplasma* spp., *Bartonella* spp.), systemic fungal diseases, and granulomatous infectious lymphadenitis (e.g., mycobacteriosis or brucellosis). The dog underwent a standard diagnostic protocol based on full blood count, biochemical profile, serum protein electrophoresis, urinalysis, a pet-side test providing comprehensive vector-borne disease screening (Idexx Europe B.V., LR Hoofddorp, The Netherlands), and *Leishmania infantum* screening (Idexx Europe B.V.); in addition, smears obtained from popliteal and submandibular lymph nodes were stained with Romanowsky stain (Merck KGaA, Darmstadt, Germany). Diagnostic imaging was also performed to evaluate the presence of visceral lesions: thoracic radiographs (3-view thorax: right lateral, left lateral, ventrodorsal views) and abdominal ultrasonography. Following initial fine-needle aspiration (FNA) cytology of the popliteal and submandibular lymph nodes, which revealed intracellular unstained bacterial rods consistent with mycobacteria, an excisional biopsy of the right popliteal lymph node was performed. This procedure was required to obtain an adequate amount of tissue for histopathological confirmation (assessment of granulomatous lesions and Ziehl–Neelsen staining), high-yield bacterial culture, and molecular subspecies identification, while minimizing potential zoonotic risks associated with sample handling. The specimen was submitted to Istituto Zooprofilattico Sperimentale of Piemonte, Liguria and Valle D’Aosta (IZSPLV) in Turin (Italy).

**Histopathology.** For histopathological examination, a representative portion of the popliteal lymph node was collected and immediately fixed in 10% neutral buffered formalin for routine tissue preservation and processed according to standard paraffin-embedding procedures. Briefly, following fixation, the tissue was dehydrated through graded ethanol solutions, cleared in xylene, embedded in paraffin wax, and sectioned at a thickness of 4 ± 2 μm using a rotary microtome. The sections were mounted on glass slides and stained with hematoxylin and eosin (H&E) for routine histopathological evaluation. To investigate the presence of acid-fast microorganisms, additional serial sections were stained using the Ziehl–Neelsen method (ZN). Prior to carbol-fuchsin staining, sections underwent a pre-treatment with 1% periodic acid solution to enhance the visualization of acid-fast bacilli within tissue sections. A bovine lymph node, previously confirmed as positive for *Mycobacterium bovis* by mycobacterial culture, was processed in parallel and used as a positive control to verify performance and reliability of the staining procedure. Histopathological assessment was performed by experienced veterinary pathologists using a light microscope. All stained sections were systematically examined at progressively increasing magnifications (10×, 20×, and 40× objectives) to evaluate tissue architecture, inflammatory changes, granulomatous lesions, necrosis, mineralization, and the possible presence, distribution, and morphology of acid-fast bacilli.

**Mycobacteria detection.** A portion of the popliteal lymph node was sampled and, after removal of fat and connective tissue, was subjected to shredding and blending using a Stomacher^®^ 400 Circulator (VWR International srl, Milano, Italy); two aliquots of the resulting homogenate were decontaminated using two different protocols: the first based on 2% sodium hydroxide (NaOH, Sigma-Aldrich Laborchemikalien GmbH, Seelze, Germany) and the second based on 1.5% hexadecylpyridinium chloride monohydrate (HPC, Applichem Services, Darmstadt, Germany). Each aliquot was centrifuged and then inoculated onto three selective solid media: Stonebrink Medium, Lowenstein Jensen Medium, and Lowenstein Jensen w/o glycerin Medium (Microbiol & C. snc, Cagliari, Italy). Tubes were incubated for the first 10 days at 37 °C in a modified atmosphere with the addition of 5% CO_2_ and for the remaining period (50 days) at 37 °C aerobically. The sample was also tested by VersaTREK/ESP Culture System II (Thermo Fisher Scientific, Waltham, MA, USA), a liquid culture method that uses bottles containing cellulose sponges that increase the surface area exposed to headspace oxygen, with a unique 7H9-based growth medium matrix enriched with specific growth factors and an antibiotic supplement. In this system, early growth is detected by automatic monitoring of changes in gas production and consumption associated with microbial growth in the inoculated bottle. Suspected colonies grown on solid media were inactivated at 95 °C for 30 min, and DNA was extracted by heat treatment and used in a multiplex polymerase chain reaction (PCR) assay, using a modified method [17,18], to identify the acid-fast bacilli. To differentiate among the MAC members, isolates were subjected to further analysis to detect both IS901 (143 bp) and IS1245 regions (472 bp), to differentiate between *M. avium* subspecies *avium* (MAA) and *M. avium* subsp. *hominissuis*. MAA is characterized by the presence of 2–17 copies of the *IS*901 insertion sequence and a single copy of *IS*1245. MAH does not possess *IS*901 and is characterized by 7–27 copies of *IS*1245. Two different PCR protocols were performed using two specific primer pairs: one targeted to IS901 [IS901se (5′-GTGATCAAGCACCTTCGGAA-3′) and *IS*901pe (5′-GCTGCGAGTAGCTTGATGAG-3′)] and one targeted to *IS*1245 [*IS*1245 P1 (5′-GCCGCCGAAACGATCTAC-3′) and *IS*1245 P2 (5′-AGGTGCCGTCGAGGAAGAC-3′)] [19]. Reaction products were electrophoresed on a 2% agarose gel containing GelRed™ Nucleic Acid Gel Stain, 10,000× in Water (Thermo Fisher Scientific) and detected by UV transillumination (Geldoc BIO-RAD, Hercules, CA, USA).

**Determination of the Minimum Inhibitory Concentration.** Susceptibility testing was performed using the Sensititre™ SLOWMYCO microdilution panel (Thermo Fisher Scientific, Waltham, MA, USA) following standard broth microdilution procedures and guidelines for nontuberculous mycobacteria (NCCLS 2003; CLSI M24-M62) [20,21]. The panel evaluated minimum inhibitory concentrations (MICs) for clarithromycin, moxifloxacin, amikacin, ciprofloxacin, doxycycline, ethambutol, linezolid, rifabutin, rifampin, and trimethoprim/sulfamethoxazole. Interpretive criteria and breakpoints for *Mycobacterium avium* complex (MAC) were assigned based on CLSI guidelines and reference literature [22,23,24]. Quality control was routinely monitored using the reference strain *Mycobacterium avium* ATCC 25291.

## 3. Results

At the first clinical examination, the dog showed cachexia (weight: 19.3 kg; body condition score: 2/5), hyperthermia (39.8 °C), and generalized lymphadenopathy, especially involving submandibular and prescapular lymph nodes.

Microcytosis, anisocytosis, acanthocytosis, severe leukocytosis with a predominance of segmented neutrophils, and monocytosis (Table 1) were recorded. The biochemical profile showed an inflammatory pattern with hypoalbuminemia, increased C-reactive protein, and decreased total iron-binding capacity (TIBC) and total iron. The serum protein electrophoresis confirmed a marked reduction in relative albumin (35.2%, reference interval [RI]: 53.1–66.0%), which was consistent with severe absolute hypoalbuminemia (2.2 g/dL, RI: 2.7–3.6 g/dL) on serum biochemistry. An increase in alpha- (27.9%, RI: 11.7–17.3%) and beta-globulins (26.6%, RI: 10.2–20.1%) was also observed. The urinalysis was unremarkable, and tests for tick-borne diseases and *Leishmania infantum* were negative.

Lymph node cytology revealed small, mature lymphocytes and a marked inflammatory component (segmented neutrophils, macrophages, and epithelioid cells) on a bloody background. The macrophages showed frequent phagocytosis of non-staining bacterial rods, suggesting mycobacterial infection (Figure 1a).

This preliminary cytological finding prompted the immediate initiation of an empirical broad-spectrum multidrug protocol comprising doxycycline (10 mg/kg orally q12h) [25], rifampicin (15 mg/kg orally q24h), and enrofloxacin (5 mg/kg orally q24h), alongside anti-inflammatory and gastroprotective therapy with meloxicam (0.1 mg/kg orally q24h) and omeprazole (0.7 mg/kg orally q24h), to target common intracellular infectious agents while awaiting definitive mycobacterial isolation.

Diagnostic imaging was performed to evaluate potential visceral lesions due to mycobacteria. Baseline thoracic radiographs (3-view thorax: right lateral, left lateral, and ventrodorsal views) were unremarkable (Figure 2a). However, because acute clinical distress marked by dyspnea and tachypnea developed two weeks later, repeat diagnostic imaging was performed to evaluate thoracic disease progression. Thoracic computed tomography (CT) revealed abundant bilateral pleural effusion, moderately enlarged cranial mediastinal lymph nodes, and moderate thickening of the pleura (Figure 2c). Abdominal ultrasonography revealed marked hepatomegaly and splenomegaly with homogeneous echogenicity, as well as generalized enlargement, globular shape, and reduced echogenicity of the mesenteric lymph nodes; moreover, the mesenteric fat appeared increased in volume, and free fluid between the bowel loops was detected.

Histologically, all slides were considered positive for the presence of acid-fast rods within granulomatous lesions. The popliteal lymph node tissue was extensively and diffusely replaced by severe granulomatous lymphadenitis, resulting in an almost complete loss of the normal lymphoid architecture. The lesions consisted predominantly of densely packed macrophages and epithelioid macrophages, intermingled with multifocal neutrophilic infiltrates and supported by abundant fibrous connective tissue (Figure 1b).

ZN staining revealed numerous intracellular acid-fast bacilli within the cytoplasm of macrophages and epithelioid cells, frequently present in large aggregates (Figure 1c), confirming a high intralesional mycobacterial burden.

Regarding microbiological diagnostic outcomes, suspected colonies grew in 5 days on VT System and in 10 days on conventional solid media.

The isolate, initially identified as *M. avium*, was subsequently typed as *Mycobacterium avium* subsp. *hominissuis* following the identification of IS1245 and the lack of *IS*901.

The MAH isolate was susceptible to clarithromycin (MIC < 0.06 µg/mL) and moxifloxacin (MIC = 0.5 µg/mL).

Two weeks after the initial presentation, repeat diagnostic imaging was clinically indicated due to the acute onset of respiratory distress (dyspnea and tachypnea at 57 breaths/min) to evaluate for intrathoracic disease progression. Follow-up thoracic radiography demonstrated moderate pleural effusion (Figure 2b), which was further characterized by thoracoabdominal CT. Ultrasound-guided thoracentesis yielded approximately 2100 mL of white, turbid fluid with continuous daily production. The fluid was not submitted for further analysis because MAH systemic infection had already been confirmed. The blood count showed hypochromic microcytic anemia, marked leukocytosis with a left shift, and monocytosis (Table 1). The biochemical profile indicated worsening of the initial inflammatory reaction, with hypoproteinemia, hypoalbuminemia, and decreases in transferrin, unsaturated iron-binding capacity (UIBC), and total iron-binding capacity (TIBC). In addition, there was an increase in ferritin (352 ng/mL, RI 85–286) and C-reactive protein (7.03 mg/dL, RI 0.01–0.22). The urinalysis and the coagulation profile were unremarkable, except for elevated fibrinogen (555 mg/dL, RI 152–284). Computed tomography (CT) of the thoracic cavity again showed abundant bilateral pleural effusion, moderately enlarged cranial mediastinal lymph nodes, and moderate thickening of the pleura (Figure 2c). Moreover, abdominal CT again showed enlargement of the liver and mesenteric lymph nodes, in addition to peritoneal thickening and nonspecific enteropathy.

The antibiotic therapy was promptly amended according to the MIC test results. Clarithromycin (5 mg/kg orally every 12 h) and moxifloxacin (10 mg/kg orally every 24 h) were initiated on Day 21, approximately two weeks after the removal and biopsy of the popliteal lymph node. Approximately four weeks after the initiation of MIC-guided therapy (Day 50), complete blood count and serum biochemistry showed a progressive trend toward normalization of red cell mass and serum proteins. C-reactive protein (CRP) remained transiently elevated (9.91 mg/dL), consistent with ongoing inflammatory activity during clinical recovery. By Day 71, approximately 50 days after initiation of MIC-guided therapy, the dog was in good clinical condition, with complete resolution of the pleural effusion and normalization of the evaluated hematological and inflammatory parameters, including CRP (0.15 mg/dL) (Table 1). No clinical relapse was reported during the available follow-up period. Because no additional biological samples were submitted after treatment, microbiological eradication could not be confirmed.

No clinical relapse was reported during the available follow-up period. No additional biological samples were submitted after treatment; therefore, microbiological eradication could not be confirmed.

A comprehensive chronological overview of clinical evaluations, diagnostic procedures, therapeutic modifications, and follow-up outcomes is summarized in Table 2.

## 4. Discussion

Before 1990, only limited information was available on MAC infections in pets. Dogs were considered resistant to *M. avium* infection [24], although a breed predisposition has been suggested for Miniature Schnauzer and Basset Hound dogs [26]. When a young dog (less than 4 years of age) belonging to a predisposed breed presents with nonspecific clinical signs such as weight loss, lethargy, and generalized lymphadenopathy, or with a history of chronic diarrhea and inappetence [15], MAC infection should be included among the differential diagnoses. In the Basset Hound breed, an X-linked combined immunodeficiency, recessively inherited, has been reported. This defect has been characterized by lymphopenia, depressed T cell function, low IgG and IgA concentrations and variable IgM concentrations [11]. Usually, this condition causes death in male puppies before four months of age. Although the age of the dog in the present case is not consistent with this condition, an underlying immunodeficiency could not be completely excluded.

In most reported cases [5,7,9,10,11,12,27], MAC-infected dogs were euthanized following rapid and progressive clinical deterioration. In contrast, the present case illustrates the potential value of an integrated diagnostic approach in selected companion animals, allowing treatment to be considered even in a severe and disseminated infection. This approach may contribute to improving clinical management while supporting animal welfare and preservation of the human–animal bond. Reliable and rapid disease identification and an effective antimicrobial treatment regimen are priorities, especially for a potentially zoonotic agent such as MAC, whose prevalence in humans has increased worldwide and which accounts for approximately 80% of nontuberculous mycobacterial infections in humans [14,16].

MAC infections in dogs pose epidemiological challenges because of the ubiquitous distribution of these organisms, their broad host range, the nonspecific nature of clinical signs, and the potentially long incubation period [24].

The apparently low incidence of MAC infections in dogs may be partly explained by the fact that avian tuberculosis is considered quite rare in dogs [24]. Consequently, MAC infection may be underdiagnosed and insufficiently considered in dogs presenting with granulomatous lymphadenitis or other compatible clinical manifestations [27].

Although the source and timing of infection often remain unknown, MAC infection should be considered in dogs with chronic granulomatous enteric disease, particularly when there is a history of close contact with poultry or swine [13], predation on wild animals, or consumption of raw food. To the best of the authors’ knowledge, this is the fourth reported case of systemic MAH infection in a dog in Italy since 2011 [15,28,29] and one of the few detailed reports describing clinical and clinicopathological recovery following culture- and MIC-guided combination therapy. The relatively short follow-up period is a limitation of this case report. Successful management of disseminated MAC/MAH infection in dogs remains exceptionally rare.

The literature indicates that most affected dogs die or are euthanized due to severe progressive deterioration or recurrence of multidrug-resistant strains several months after the initial MAH diagnosis [29]. Reports of clinical recovery from confirmed MAH infection in canine patients treated with a targeted regimen are extremely limited in the veterinary literature; the favourable outcome achieved in this case highlights the critical importance of transitioning from broad empirical coverage to susceptibility-guided antimicrobial therapy when microbiological identification and susceptibility testing are available.

Several complications secondary to MAH infection may further compromise the clinical condition, including chylothorax resulting from lymphatic obstruction or dysfunction. In the present case, the white, turbid fluid with continuous daily production observed two weeks after the initial presentation may have been associated with compression or dysfunction of the thoracic lymphatic system. However, because the fluid was not subjected to specific laboratory analysis, a chylous origin could not be confirmed. This represents a limitation of the case report, although the result of such an analysis would not have altered the antimicrobial treatment or the clinical management. The management of disseminated MAC infection requires a transition from empirical treatment to culture- and susceptibility-guided targeted therapy whenever possible. Monotherapy or empirical regimens using older fluoroquinolones (such as enrofloxacin) or tetracyclines (such as doxycycline) often fail to achieve clinical resolution due to intrinsic resistance mechanisms in *M. avium* complex isolates [25]. Optimal NTM treatment relies on a targeted combination of drugs, such as a potent macrolide (e.g., clarithromycin) combined with newer-generation fluoroquinolones (e.g., moxifloxacin or pradofloxacin) or rifampicin [25]. In the present case, upon definitive isolation of MAH and determination of MIC values, the initial empirical protocol was promptly adjusted. Following definitive isolation of MAH and determination of the MIC values, the initial empirical protocol was promptly adjusted. Clarithromycin (MIC < 0.06 µg/mL) and moxifloxacin (MIC = 0.5 µg/mL) were selected based on the in vitro susceptibility profile. This susceptibility-guided combination was followed by marked clinical and clinicopathological improvement, including complete resolution of the pleural effusion and normalization of the evaluated hematological and inflammatory parameters during the available follow-up period. However, microbiological eradication was not confirmed [11].

## 5. Conclusions

Prompt strain identification and the availability of antimicrobial susceptibility testing can support the selection of an appropriate targeted antimicrobial regimen and may help reduce unnecessary exposure to multiple antibiotic agents [4,6,8]. In the present case, multidisciplinary and novel diagnostic approaches were integrated with complete follow-up based on diagnostic imaging, blood counts, and biochemical profiles until abnormal parameters returned to normal. A longer follow-up period of at least twenty-four months should be considered, as suggested by Marianelli et al. [28], to avert the emergence of drug-resistant clones. As reported in HIV-positive patients with MAH infection, immune deficiency could promote a more severe outcome, requiring prolonged antibiotic treatment, clinical follow-up, and specialist investigations.

## Figures and Tables

**Figure 1:**
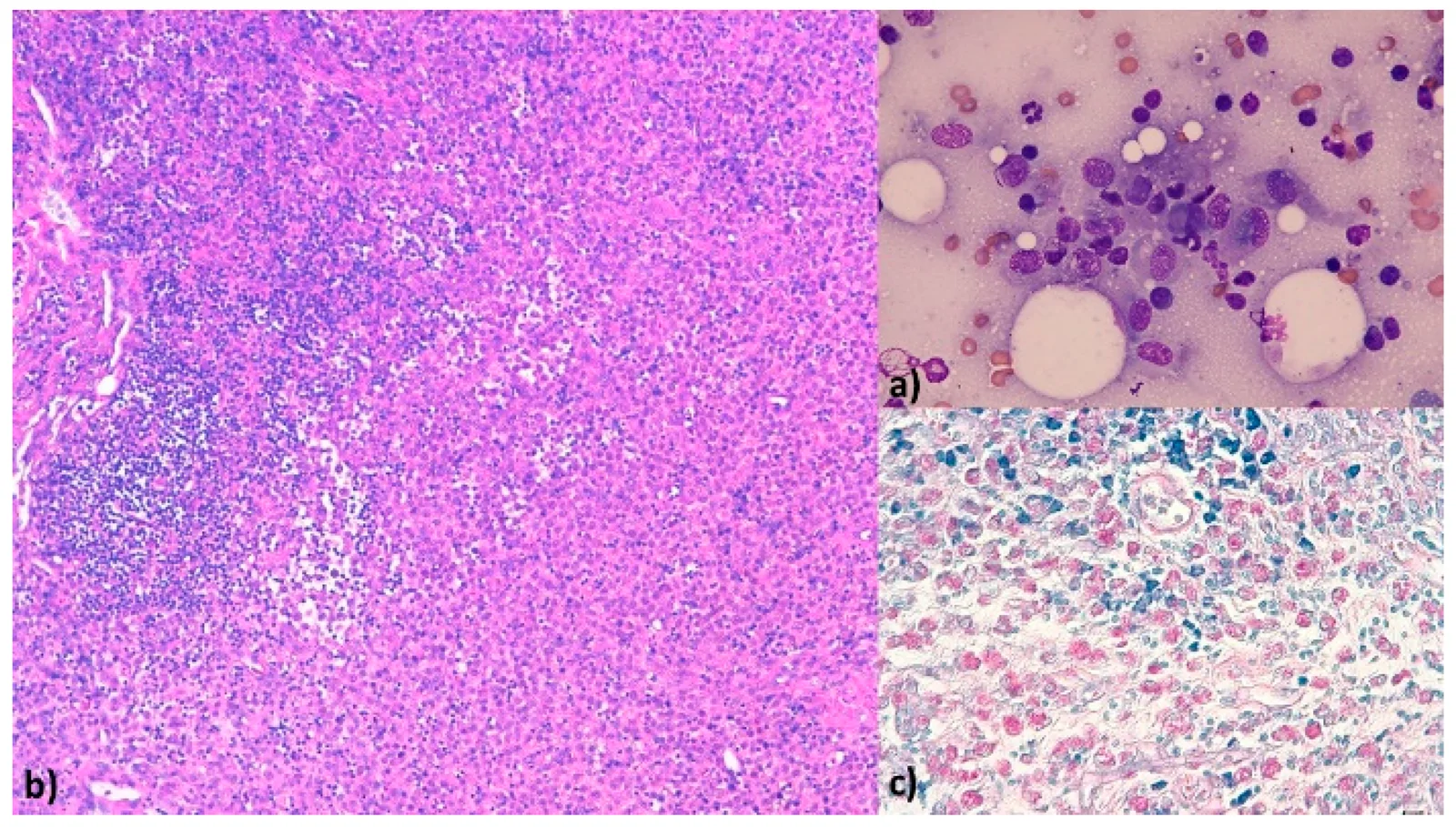
Cytological, histopathological, and histochemical findings in the popliteal lymph node. (**a**) Cytological lymph node smear showing mature lymphocytes intermingled with a massive inflammatory infiltrate composed of neutrophils, macrophages, and epithelioid cells. Macrophages exhibit cytoplasm filled with non-staining bacterial rods (negative ghosts). Romanowsky stain; 100× objective. (**b**) Histopathological section of popliteal lymph node demonstrating severe, diffuse granulomatous lymphadenitis with loss of normal architecture and massive infiltration of macrophages and epithelioid cells. Haematoxylin and Eosin (H&E) stain; scale bar = 100 µm. (**c**) High-magnification Ziehl–Neelsen (ZN) stain under oil immersion (100× objective) highlighting abundant intracellular acid-fast bacilli (indicated by black arrowheads) arranged in dense aggregates within the cytoplasm of macrophages and epithelioid cells. Scale bar = 10 µm.

**Figure 2:**
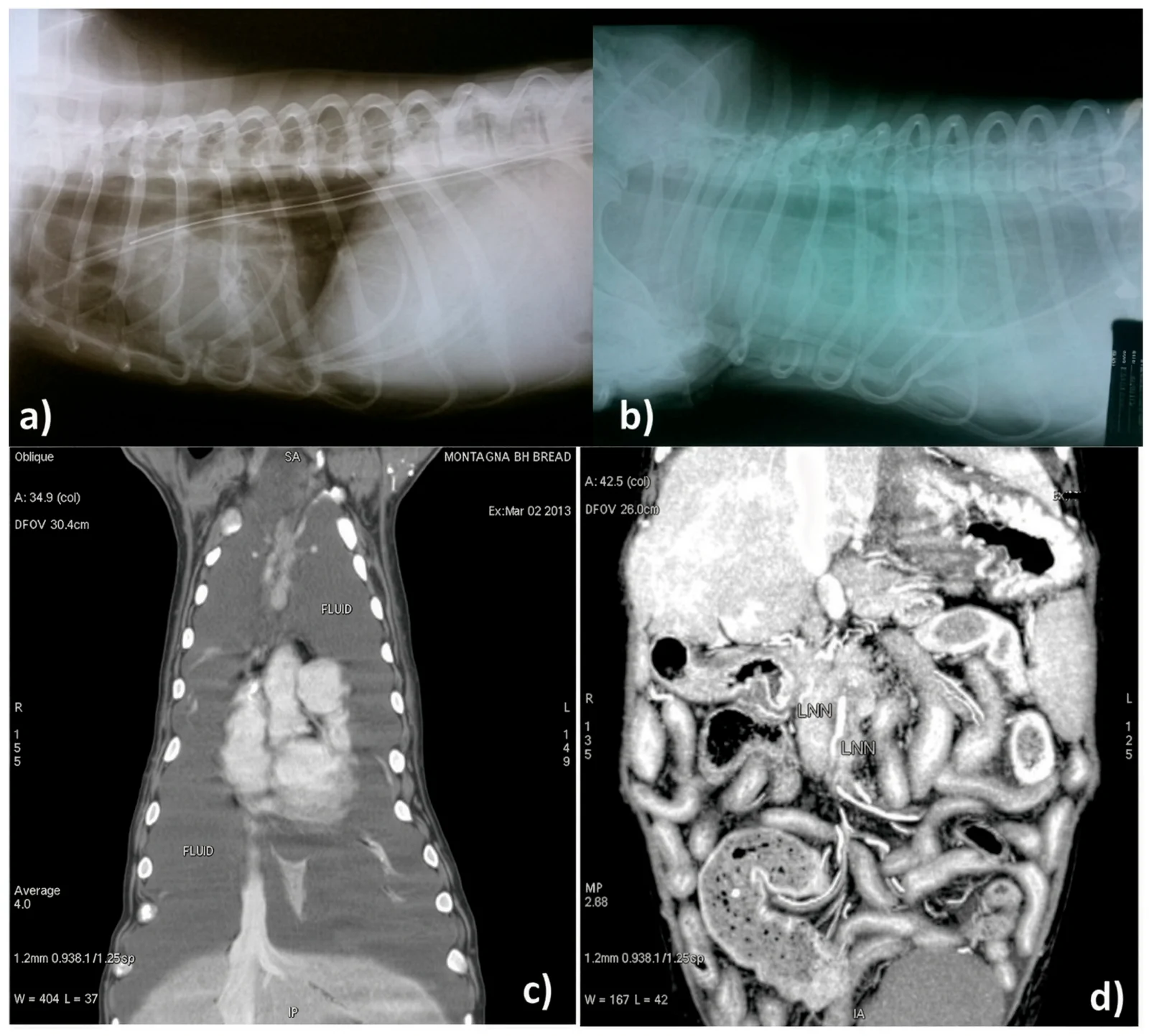
Diagnostic imaging findings at different stages of disease. (**a**) Baseline left lateral thoracic radiograph showing no significant parenchymal abnormalities. (**b**) Follow-up left lateral thoracic radiograph (2 weeks later) demonstrating moderate pleural effusion. (**c**) Thoracic computed tomography (CT) scan (transverse view) displaying abundant bilateral pleural effusion, thickening of the pleura, and cranial mediastinal lymph adenomegaly. (**d**) Abdominal computed tomography (CT) showing marked hepatomegaly, splenomegaly, and enlarged, rounded, hypoechoic mesenteric lymph nodes surrounded by hyperechoic reactive mesenteric fat and free abdominal fluid.

**Table 1:** Variations in hematological and biochemical parameters at different time points during the clinical course (Day 0, Day 14, Day 21, Day 50, and Day 71).

Parameter	Unit	Reference Range	Onset (Day 0)	2 Weeks Later (Day 14)	Post-Treatment Change (Day 21)	1 Month Post-Change (Day 50)	Final Follow-Up (Day 71)
HCT	%	39.0–59.2	34.1	31.40	36.9	45.5	48.2
RBC	×10^6^/µL	5.70–8.56	5.12	4.9	5.38	6.72	7.1
Hb	g/dL	14.1–21.2	11.8	11	12.1	13.8	15.2
WBC	×10^3^/µL	5.45–12.98	27.20	33.70	29.1	27.73	9.8
Neutrophils	×10^3^/µL	3.55–9.31	22.57	28.82	25.1	24.57	6.4
Monocyte	×10^3^/µL	0.19–0.80	2.18	2.06	1.45	1.05	0.42
Total Protein	g/dL	5.7–7.1	5.1	3.5	6	6.4	6.5
Albumin	g/dL	2.7–3.6	2.2	1.3	2.3	2.8	3.1
CRP	mg/dL	0.01–0.22	13.28	7.03	13.54	9.91	0.15
Ferritin	ng/mL	85–286	-	352	-	210	145
TIBC	µg/dL	318–479	241	156	305	390	412
UIBC	µg/dL	182–306	-	122	221	245	260
Iron (Fe)	µg/dL	95–226	50	34	84	120	142

Abbreviations: HCT, Hematocrit; RBC, Red Blood Cell Count; Hb, Hemoglobin; WBC, White Blood Cell Count; CRP, C-reactive protein; TIBC, Total Iron-Binding Capacity; UIBC, Unsaturated Iron-Binding Capacity.

**Table 2:** Chronological summary of clinical presentation, diagnostic steps, therapeutic interventions, and clinical outcomes.

Time Point	Clinical Findings	Diagnostic Procedures & Results	Therapeutic Interventions
Day 0	Lethargy, anorexia, weight loss (BCS 2/5), fever (39.8 °C), generalized lymphadenopathy, shifting lameness.	Bloodwork: leukocytosis, monocytosis, microcytosis, low albumin, elevated CRP. Diagnostic Imaging: abdominal hepatosplenomegaly & lymph adenomegaly. Lymph Node Cytology: intracellular non-staining rods.	Initiated Empirical Protocol: - Doxycycline (10 mg/kg q12h) - Enrofloxacin (5 mg/kg q24h) - Rifampicin (15 mg/kg q24h) - Meloxicam & Omeprazole
Day 7	Popliteal lymph node biopsy performed.	Histopathology: severe granulomatous lymphadenitis. ZN Stain: abundant acid-fast bacilli. Bacterial Culture & PCR initiated.	Continued empirical protocol.
Day 14	Dyspnea, tachypnea (57 bpm).	Thoracic Radiography & CT: moderate/severe bilateral pleural effusion. Thoracentesis: 2100 mL turbid fluid evacuated.	Pleural drainage performed. Continued empirical protocol.
Day 21	Diagnostic confirmation & MIC results available.	Culture/PCR: *M. avium* subsp. *hominissuis* (MAH). MIC Panel: Sensitive to Clarithromycin (<0.06 µg/mL) and Moxifloxacin (0.5 µg/mL).	Therapy Optimization (MIC-guided): Discontinued Doxycycline & Enrofloxacin. Started Clarithromycin (5 mg/kg q12h) & Moxifloxacin (10 mg/kg q24h).
Day 50	Clinical improvement; respiratory effort normalized. Hematological and biochemical parameters showed a progressive trend toward normalization	Bloodwork: trend toward normalization of WBC and proteins; persistent mild CRP elevation.	Continued targeted therapy.
Day 71 (Follow-up)	Complete clinical and clinicopathological recovery; body weight gain and normal vitality.	Diagnostic imaging showed complete resolution of pleural effusion and lymphadenomegaly. Hematological and inflammatory parameters returned to within reference intervals.	Continued clinical monitoring.

## Data Availability

The data presented in this study are available from the corresponding author upon reasonable request. The data are not publicly available as they are stored in the institution’s internal clinical records system.

## Abbreviations

The following abbreviations are used in this manuscript:

H&E	Hematoxylin and Eosin staining
MAA	*Mycobacterium avium* subspecies *avium*
MAC	*Mycobacterium avium* complex
MAH	*Mycobacterium avium* subspecies *hominissuis*
MIC	Minimum Inhibitory Concentration
NTM	Nontuberculous Mycobacteria
VT	VersaTREK
ZN	Ziehl-Neelsen staining
